# The Tunnelled Atrial Catheter: A Promising Solution for Vascular Capital Depletion in Dialysis despite Associated Thrombi

**DOI:** 10.1155/2024/5219914

**Published:** 2024-03-15

**Authors:** Meriam Hajji, Salah Saied, Ikram Mami, Yassine Khadhar, Tasnim Ben Ayed, Imen Gorsane, Fethi Ben Hamida, Jalel Ziadi, Mohamed Karim Zouaghi, Ezzeddine Abderrahim

**Affiliations:** ^1^Department of Medicine A, Charles Nicolle Hospital, Tunis, Tunisia; ^2^Laboratory of Kidney Pathology LR00SP01, Tunis, Tunisia; ^3^Faculty of Medicine of Tunis, University of Tunis El Manar, Tunis, Tunisia; ^4^Department of Nephrology, La Rabta Hospital, Tunis, Tunisia; ^5^Department of Cardiac and Vascular Surgery, La Rabta Hospital, Tunis, Tunisia

## Abstract

**Introduction:**

Longer survival in dialysis led to a higher incidence of vascular access complications and failure. With the limited access to kidney transplantation programs and peritoneal dialysis, exhaustion of vascular access for hemodialysis is an increasingly common situation. Among the available options, atrial tunneled dialysis catheter (ATDC) has been reported as an effective vascular access in this population. *Methodology*. We report the experiences of two nephrology centers in Tunis with ATDC as an ultimate vascular access for dialysis. *Case Reports*. Two patients with exhausted vasculature underwent ATDC insertion in 2020 and 2022, respectively, as a vascular access of last resort. Both patients underwent CRBI, which resolved with favorable outcomes. One case was complicated by post-operative thrombosis and was successfully treated with thrombolysis. Both patients are currently on dialysis via their ATDC with a catheter patency of 29 months.

**Conclusion:**

ATDC is a life-saving and safe vascular access in cases of depleted vasculature. Little more than 50 cases have been reported in the literature during the last 30 years. As the frequency of vasculature exhaustion is expected to increase, preservation of veinous access in patients at risk of chronic kidney disease have never been more crucial.

## 1. Introduction

The advancement of care standards for end-stage renal disease (ESRD) and the growing number of patients with chronic kidney disease (CKD) in need of renal replacement therapy (RRT) have contributed to a prolonged survival of hemodialysis patients. With kidney transplantation (KT) often not an option, vascular access complications and failures are becoming increasingly common, resulting in a growing population of ESRD patients with depleted vasculature. In such dramatic situations, the challenge is not only to implement a last resort vascular access, usually a catheter, in an unusual site but also to preserve it. In this multicentered study, we report the cases of two patients with an exhausted vasculature in whom an atrial tunneled dialysis catheter (ATDC) was inserted respectively in 2020 and 2022, respectively. A catheter-related bloodstream infection (CRBI) occurred in both cases with favorable outcomes while thrombosis complicated the early post-operative period in one case successfully treated with thrombolysis as a salvage therapy.

## 2. Methods

First, we recorded the data of both patients, respectively followed in the nephrology department of Charles Nicolle Hospital and La Rabta Hospital in Tunis in whom a ATDC was inserted in 2020 and 2022, respectively, and the outcomes of the procedure. Then, a search for similar case reports was performed in PubMed. We searched for publications with the following key words “exhausted vascular access,” “atrial vascular access for hemodialysis,” “hemodialysis catheter thrombolysis,” “right atrial dialysis catheter thrombosis,” “lock solutions hemodialysis” and later discussed the ATDC outcomes in situations of vasculature exhaustion in patients with ESRD.

## 3. Results

### 3.1. Cases Presentation

#### 3.1.1. Case 1

**Figure 1:**
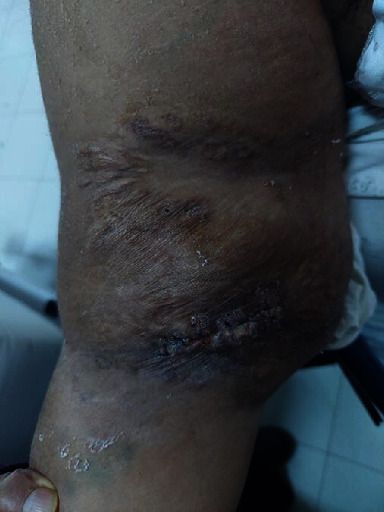
Thrombosed humeral graft in the upper right limb.

**Figure 2:**
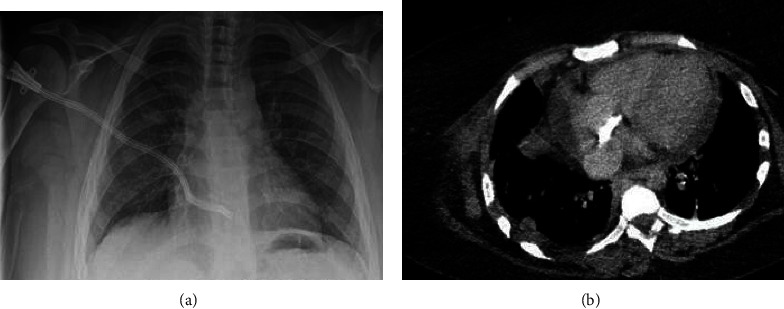
(a) Chest radiography showing the ATDC. (b) CT-scan showing the catheter location at the right atrium.

**Figure 3:**
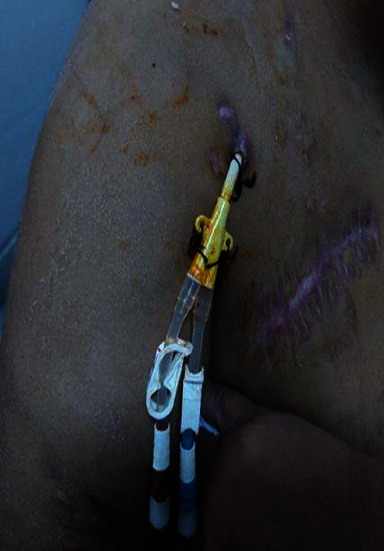
Catheters' exit-site.

Our patient was a 32-year-old female with a history of chronic kidney disease due to chronic tubulo-interstitial nephropathy. A diagnosis of Senior-Loken syndrome is highly suspected due to the patient's associated congenital and bilateral blindness in a context of first-degree parental consanguinity. After a 4-year gap in her follow-up, she presented at our center in end-stage renal disease requiring renal replacement therapy. Peritoneal dialysis was not considered due to the patient's morbid obesity (BMI = 45 kg/m^2^), blindness, and precarious socio-economic situation. Kidney transplantation was not an option as no donor was available. Hemodialysis was initiated via a left cephalic fistula. After 87 months of dialysis, the patient had experienced at least 6 vascular access complications, all resulting in thrombosis (Figure 1). An antithrombin III deficiency was diagnosed, and the patient was treated with long-term vitamin K antagonists (VKA). After her right axillary graft thrombosis, a thrombectomy via a Fogarty catheter failed and the patient was addressed to our center. A vascular cartography showed multiple stenosis and signs of acute and chronic thrombosis in all deep and central veins, making impossible any attempt to place a new catheter. Our patient survived 12 days without dialysis with a strict dietary and fluid intake before the ATDC was finally placed as a last resort vascular access by the heart surgery team. The intervention was performed under general anaesthesia, with the patient in a supine position. An anterior right thoracotomy was done at the second right intercostal space. After opening the pericardium, the right atrium was directly punctured under visual control. A tunneled catheter of 40 centimeters of length was then placed in this atrium and attached to the skin (Figures 2 and 3). After purging the catheter, haemostasis of all tissues was done and the closing of the thoracotomy was performed plan by plan, by setting up a thoracic drain which was maintained in aspiration.

The post-operative course was marked by a CRBI with no identifiable microorganisms found in both the catheter and peripheral blood cultures. Treatment with a three-week course of broad-spectrum antibiotics led to favorable outcomes. It is noteworthy that anticoagulation therapy with VKA was temporarily discontinued after catheter placement due to the presence of a circumferential pericardial effusion but was reinstated one week later after multiple and prolonged hemodialysis sessions and a trans-thoracic echocardiography showed no evidence of the effusion. Two weeks after catheter placement, a low flow in both catheter lumens was noted, and the dialysis session was interrupted due to the absence of blood flow in both streams. The failure of aspiration and flush through the catheter and a wire guide failure to pass through the venous lumen further suggested thrombosis. Thrombolysis using Alteplase was performed by the cardiac surgery team under local anesthesia. The procedure began with a systemic injection of a heparin bolus at a dose of 0.5 milligrams per kilogram. An opacification was then performed by injecting iodinated contrast product via the tunneled catheter, which confirmed a thrombosis of the latter a few centimeters before its insertion in the cardiac cavity with a patent distal end. A hydrophilic guidewire of 0.035 inches in diameter and 150 centimeters in length was introduced via the tunneled catheter and guided towards the right atrium under scopic control. A five French Cobra catheter was then introduced over the guidewire and placed on the right atrium, with the guidewire being removed while taking care to keep the Cobra catheter in place, all under scopic control. The Alteplase solution, consisting of a 50 milliliters vial of solvent mixed with a 50 milligrams vial of Alteplase powder, was injected via the Cobra catheter, first in the right atrium, then along the lumen of the tunneled catheter, by gradually withdrawing the Cobra catheter under scopic control. The total quantity injected was 50 milligrams of Alteplase, equivalent to 29 million international units (MUI). After a three-day dwell time, the catheter was functional again, allowing the patient to start dialysis sessions once again. A heparin lock-solution was used systematically at the end of each session, and the patient was discharged after a week of observation.

#### 3.1.2. Case 2

A 22-years-old male patient followed from a young age for an ESRD secondary to a chronic tubulo-interstitial nephropathy complicating a CAKUT (ureteropelvic-junction obstruction and vesicoureteral reflux) was referred to the nephrology department of La Rabta Hospital at the age of 20 for an exhausted vasculature, 53 months after dialysis start. During this period, numerous tunneled and non-tunneled central venous catheters were inserted and a total of two AVFs and three arterio-venous grafts (AVGs) were created, all complicated with thrombosis. CT-venography revealed a chronic thrombosis in both subclavian and femoral veins respectively extended to the superior and inferior vena cava. Further investigations found no evidence of a congenital or acquired thrombophilia. At first, peritoneal dialysis (PD) was chosen as a RRT. Later, due to a menacing hyperkalemia and volume overload, a tunneled femoral catheter (TFC) was emergently inserted by surgical mean and the patient started a regimen combining PD and once-weekly hemodialysis. A year later, the TFC was removed due to a dysfunction caused by a severe CRBI. Peritoneal dialysis proved ineffective due to freent volume overload state further worsened by anuria. Ultimately, he developed a severe septicemia requiring broad-spectrum antibiotics alongside hyperkalemia, volume overload and an acute anemia. The urgent need to start hemodialysis prompted the decision to insert an ATDC as a last resort vascular access as described above. A post-operative and extended hemodialysis session was successfully performed, accompanied by a red-cell blood transfusion. One month later, the patient developed septicemia characterized by chills and fever during hemodialysis. Despite no pathogens being identified, the patient had a favorable outcome after a three-week course of broad-spectrum antibiotics. Nine months later, the peritoneal catheter was removed due to severe peritonitis. After 29 months of follow-up, the catheter remained functional with the ability to undergo three hemodialysis sessions per week and had a satisfactory blood flow. No incidents or complications have been reported since discharge from the hospital.

## 4. Discussion

**Figure 4:**
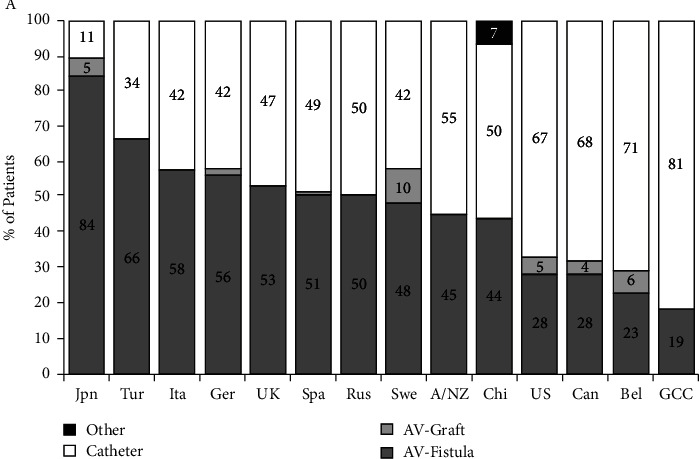
Trends in vascular access in incident hemodialysis patients across the world [2].

Renal replacement therapy consists of hemodialysis, peritoneal dialysis (PD), or kidney transplantation (KT). When both PD and KT are not suitable, hemodialysis remains the only available option and an arterio-venous fistula (AVF) is the recommended access in most patients [1]. However, functional vascular accesses remain a challenge in dialysis therapy. There is a wide disparity between different regions in the world, with dialysis often starting with a catheter (Figure 4) [2], exposing patients to long-term risks of catheter-related stenosis and thrombosis and further accelerating the decline of the vasculature [3, 4]. In Tunisia, there is no national registry on the epidemiology of vascular access in hemodialysis patients or chronic kidney disease in general. The available data comes from small studies that address the epidemiology, complications, and/or outcomes of vascular accesses for hemodialysis [5–9]. As the prevalence of chronic kidney disease (CKD) and its risk factors increase, situations of vasculature exhaustion, although rare, are expected to become more frequent [3]. This challenging situation often requires a drastic and immediate solution, leading to the use of unusual sites for catheter insertion, such as trans-lumbar, trans-renal, intra-atrial, and femoral tunneled catheters [10]. Trans-atrial dialysis catheter (ATDC) appears to be a safe and life-saving option, as shown by the low incidence of catheter-related complications and death [3].

In a systemic review of use of ATDC as last vascular access for hemodialysis conducted by Philipponnet and al, Complications were dominated by infection and dysfunction [3]. ATDC-related infections usually resolved with systemic antibiotics, but severe episodes of sepsis were reported, proving fatal in one case [3]. Interestingly, Oguz and al reported the largest number of cases of ATDC as a last resort vascular access for hemodialysis. Among the 27 patients included, no CRBI occurred. According to the author, this resulted from the use of systemic antibiotic prophylaxis during the first week following the procedure in patients at risk for endocarditis and sepsis [11]. Both our patients experienced a CRBI during the postoperative period with no identified germs, and favorably resolving with a course of broad-spectrum antibiotics. These observations rise many questions that need to be addressed in the future: What is the optimal timing of antibiotic prophylaxis start when an ATDC is inserted? Are there particular risk factors specific to this situation that can be identified?

Dysfunction is the other main concern shortening the catheter and patient's survival, caused by catheter kinking, fibrin sheath formation and/or thrombosis. When it comes to ATDCs, thrombosis seems to be an uncommon cause for dysfunction as it was reported in 2/10 cases [3, 12]. In both patients, no particular risks for thrombosis were reported [12] while in the case of our patient, an antithrombin III deficiency was discovered. In situations of multiple vascular access thrombosis, it might be accurate to check for risk factors of thrombosis, particularly for coagulopathies when a particular context of parental consanguinity or an indetermined nephropathy exists, as the patient's life depends on his ATDCs' patency. According to Kidney Disease Outcomes Quality Initiative (KDOQI), available treatment options for central venous catheter thrombosis are either pharmacological thrombolysis or mechanical intervention including catheter exchange or removal, with a low to moderate level of evidence [13]. KDOQI also recommends thrombolysis using Alteplase in case of conservative manoeuvres' failure as an alternative to the mechanical options [13]. A systematic review performed by Hilleman et al. evaluated the efficacy, safety, and cost of the recombinant tissue plasminogen activators Alteplase, Reteplase and Tenecteplase in dialysis catheter dysfunction found that Alteplase and Reteplase had higher success rates compared to Tenecteplase (81%, 88%, and 41%, respectively), with Reteplase being more cost-effective than Alteplase. The doses, number of administrations, and dwell time varied among studies. Notably, no adverse effects were reported [14]. However, one patient reported by Pereira et al. who underwent systemic thrombolysis with a TPA for suspected ATDC thrombosis suffered a fatal pericardial tamponade. It is important to note that the molecule and dose used were not specified [12]. In our case, thrombolysis was carried with alteplase administration in both catheter lumens allowed a successful thrombolysis as attested by the ability to start and finish dialysis sessions with a blood flow of 300 ml/min.

Prevention of catheter-related thrombosis, particularly in situations of vasculature exhaustion, is mandatory to avoid these scenarios. Use of lock solutions, mostly unfractioned Heparine at different concentrations, has long been a suitable option to prevent catheter-related complications, especially CRBI and thrombosis. But the occurrence of these complications while Heparin lock-solutions were used put into question the efficacy of Heparin solutions', thus, prompting the search for other agents that can achieve, at least, the same efficacy with a lower cost and rate of complications. Many agents have been tested through the years, yet, the optimal composition, dose, associations and rate of administration of lock-solutions are still to be determined. Wang and al also noted that rTPA were the only agents that had a benefit over heparine when used as lock solutions, although, data concerning its use as a prophylactic lock solution was limited [15].

Finally, our cases were unique due to the younger age of both patients compared to those reported in the literature, and the insertion of a trans-atrial dialysis catheter (ATDC) in a septic situation in one case that turned out to be life-saving.

## 5. Conclusion

The increasing prevalence of patients with ESRD who require dialysis and limitations in access to kidney transplantation or peritoneal dialysis mean that an increasing number of dialysis patients will eventually face vasculature exhaustion. The use of ATDC as a last resort access for dialysis has been shown to be safe and lifesaving, although complications such as infection and dysfunction have been reported and can be dangerous and even fatal. As healthcare providers, particularly nephrologists, it is important to prioritize the preservation of patients' venous access, especially for those who are at high risk or already have CKD, and to promptly address any complications that may arise from their vascular access.

## Data Availability

The data used to support the findings of this study are included within the article.
